# Response rates of standard interferon therapy in chronic HCV patients of Khyber Pakhtunkhwa (KPK)

**DOI:** 10.1186/1743-422X-9-18

**Published:** 2012-01-14

**Authors:** Bashir Ahmad, Sajid Ali, Ijaz Ali, Sadiq Azam, Shumaila Bashir

**Affiliations:** 1 Centre for Biotechnology and Microbiology, University of Peshawar, Peshawar, Khyber Pakhtunkhwa, Pakistan; 2 Institute of Biotechnology and Genetic Engineering, Agricultural University, Peshawar, Khyber Pakhtunkhwa, Pakistan; 3 Department of Pharmacy, University of Peshawar, Peshawar, Khyber Pakhtunkhwa, Pakistan

**Keywords:** End of treatment virologic response, Interferon, Ribavirin, KPK (Khyberpakhtunkhwa), Hepatitis C, ALT (Alanin Aminotransferase)

## Abstract

**Background:**

Interferon based therapy is used to eradicate the Hepatitis C Virus from the bodies of the infected individuals. HCV is highly prevalent in Khyber Pakhtunkhwa (KPK) that is why it is important to determine the response of standard interferon based therapy in Chronic HCV patients of the region.

**Study design:**

A total of 174 patients were selected for interferon based therapy. The patients were selected from four different regions of KPK. After confirmation of active HCV infection by Real Time PCR, standard interferon with ribavirn was given to patients for 6 months. After completion of therapy, end of treatment virologic response (ETR) was calculated.

**Results:**

Out of total 174 patients, 130 (74.71%) showed ETR and 44 (25.28%) did not show ETR. In district Bunir, out of 52 patients, 36 (69.23%) showed ETR and 16 (30.79%) did not show ETR. In district Mardan, out of the total 74 patients, 66 (89.18%) were negative for HCV RNA and 8 (10.81%) were resistant to therapy. In Peshawar, out of 22, 16 (60%) were negative and 6 (40%) were positive for HCV RNA at the end of 6 months therapy. In the Federally Administered Tribal Area (FATA), out of 18 only 10 (55.5%) were negative and 8 (44.45%) were positive for active HCV infection.

**Conclusion:**

It is concluded that the response of antiviral therapy against HCV infection in chronic HCV patients of KPK province is 74.71%. The high response rate may be due to the prevalence of IFN-responsive HCV genotypes (2 and 3) in KPK.

## Background

The hepatitis C virus (HCV), a major public health problem and the leading cause of chronic liver disease has affected an estimated 180 million people worldwide [1,2]. The HCV genome is single-stranded, positive-sense RNA, approximately 9600 bp long and encodes a single polyprotein. HCV infection has reached epidemic proportions and annually more than one million new cases of infection are reported world wide. It is believed that HCV infection is more than that of hepatitis B virus infection (HBV) [3]. Besides this HCV is the leading cause of liver transplantation and organ shortage is the major associated problem. The introduction of effective therapy for the prevention of such life threatening infection is the ultimate goal. It is especially more important in underdeveloped countries, where HCV infection rate is more and most of the patients have financial problems for its treatment.

Eradication of the virus is the primitive goal of hepatitis C treatment that is sustained virologic response (SVR) which is defined as absence of HCV RNA in serum after 6 months of treatment completion and is evidenced by sensitive molecular tests, Polymerase Chain Reaction (PCR). It has been observed over the past decades that there is great improvement in the SVR rates when treatment strategies have been shifted from interferon monotherapy to combination interferon plus ribavirin [4,5].

Hepatitis C therapy started with a small trial of recombinant human interferon Alfa almost 25 years ago [6]. Interferon was selected because of its broad activities against the viruses and it was thought that it might also be active against still-undiscovered agent of non-A, non-B hepatitis. No doubt, interferon was found very active against HCV and resultant effects were decrease in the level of serum alanin aminotransferase (ALT). So far HCV was not discovered, the effects of interferon were not understood but the result of using interferon was the reduction in HCV RNA level, which led to a sustained absence of virus in a proportion of patients [7]. Ribavirin a nucleoside analogue, known to have activity against several flaviviruses had strong effects in lowering the level of aminotransferase and histologic characteristics of the liver but had little effects on serum HCV RNA levels [8]. Moreover when ribavirin was combined with interferon then it has increased sustained virologic response rate [9]. Interferon and ribavirin when given in combination for 48 weeks then sustained virologic response rates was 40-50% which is two to three times more than that obtained with interferon alone [8]. Interferon alpha (IFN-α) along with ribavirin has been widely used as a standard treatment option for patients with chronic HCV infection all over the world [10]. Pegylated interferon with Ribavirin has better response rate as compared to standard interferon [11].

In Pakistan the general concept is the use of standard interferon therapy. This is partly due to economic reasons and Pakistan Society of Gastroenterology and GI Endoscopy also favours the use of SdIF in genotype-3 [12]. Government of Pakistan is also providing only SdIF via a special Prime Minister's initiative programme against hepatitis, thus PgIF is out of reach for the majority of the patients.

Response rates of standard interferon in chronic HCV patients have never been investigated in KPK. The study had focused on the efficacy of standard interferon therapy as administered in the case of chronic HCV patients in Khyber Pakhtunkhwa province of Pakistan.

## Methodology

To evaluate the response of standard interferon combination therapy against chronic HCV infection, we selected four different regions of KPK province. The regions were districts Bunir, Peshawar, Mardan and FATA region. Through coordination with practioners and lab workers, samples were collected from the suspected patients. After initial screening with ICT and Elisa, PCR test was performed for each patient sample according to the instructions of the manufacturers (Roboscreen, Germany). Among the confirmed anti-HCV patients, only 174 patients whose PCR was positive, were selected for interferon therapy keeping in mind the exclusive therapy criteria that is, age of the selected personals (18-55 years), no co-infection associated, ALT level higher than normal, platelets and Hb levels within the accepted range, and stage of cirrhosis. We selected 52 patients from Bunir, 22 from Peshawar, 74 patients from Mardan and 18 from FATA regions.

PCR positive patients were given standard interferon combination therapy i.e. interferon alpha 2a (3MIU thrice a week) plus Ribavirin (1000-1200 mg/day) continuously for 6 months with repeated testing of ALT level and HCV RNA during and after the interferon therapy.

After completion of the 6 months long therapy, the results obtained were as. Out of total 174 patients, 130 (74.71%) were negative for HCV RNA and showing end of treatment response (ETR) while 44 (25.28%) were positive for HCV RNA and did not show ETR. In district Bunir, out of 52 patients who had completed therapy, 36 patients (69.23%) showed ETR and 16 (30.79%) did not show the ETR. In district Mardan, we found that out of total 74 patients who had taken 6 months therapy, 66 (89.18%) were negative for HCV RNA and 8 (10.81%) were resistant to therapy. In Peshawar district, out of 22, 16 (60%) were negative and 6 (40%) were positive while in FATA, out of 18 only 10 (55.55%) were negative and 8 (44.45%) were positive (Table 1).

**Table 1:** Region wise distribution of positive HCV RNA samples and their respective ETR

Districts	T. Samples	Age group	Sex		ETR+	ETR-	% ETR+	% ETR-
							
			Male	Fe-male				
Bunir	52	18-55	30	22	36	16	69.23	Bunir

Mardan	74	20-54	42	32	66	8	89.18	Mardan

Peshawar	30	22-55	18	12	18	12	60	Peshawar

FATA	18	22-53	14	4	10	8	55.55	FATA

Total	174		104/70		130	44	74.71	25.28

*ETR *(End of Treatment Response), *T *(Total)

**Figure 1:**
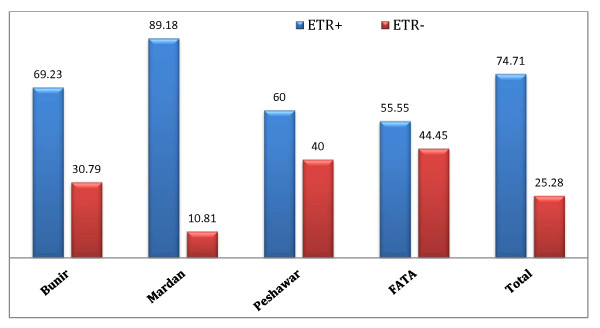
**Region wise Percent ETR Bar**.

Our study revealed that response rate of combination therapy was comparatively higher in districts Mardan (89.18%) and Bunir (69.23%) than in districts Peshawar (60%) and FATA region (55.55%). The percent response of overall therapy calculated was 74.71% (Figure 1).

## Discussion

Hepatitis C Virus infection is spreading rapidly. HCV prevalence is nearly 200 million people world wide and every year infects 3-4 million more people [13]. The seroprevalence of Hepatitis C virus in different parts of Pakistan, reported in the last 5 years, is from 2.2%-13.5%. The highest seroprevalence of hepatitis C has been reported from Lahore (13.5%) [14] Jasmshoro (9%) and Mardan (9%) [15,16].

Pakistan is a developing country and the literacy rate is also low, due to which lack of information regarding the pathogenecity, routes of transmission and the proper procedures of diagnosis and treatment are rarely followed. Therefore HCV infection has become an economic burden on the people of Pakistan and especially in KPK.

In this study we determined the End of Treatment Response (ETR), defined as the absence of HCV RNA at the end of 6 months IFN therapy, in chronic HCV patients. The average calculated ETR was 74.71% and resistance calculated was 25.28%. Different regions of KPK province had different ETR rates; like ETR was very high in district Mardan followed by district Bunir and lower in district Peshawar and FATA regions [Figure 1].

The average response rate of IFN combination therapy with ribavirin in chronic HCV patients of KPK population was 74.71% [Table 1]. Although KPK have different population groups which may vary regarding response to IFN based therapy. Instead, the response rate was similar to other studies conducted internationally [17-19] as well as locally [20,21], when treated with IFN and ribavirin combination therapy.

In district Mardan prevalence of HCV infection has been recorded as 9% [16]. The high ETR rate in district Mardan might be due to the high prevalence of HCV genotypes 2 and 3 and might be due to high literacy rate, as by adopting proper procedures for diagnosis and treatment, the efficacy rate might be increased as compared to those who have no or little knowledge.

In district Bunir HCV prevalence has also been shown to be 5% [22]. As this district is less developed and is considered as an economically poor district, through the prime minister's control programme for Hepatitis, HCV patients have been treated and ETR rate was comparatively higher than that of Peshawar and FATA regions [Table 1].

In district Peshawar the response rate was low as compared to districts Mardan and Bunir. Low response rate might be attributed to migration of people from Afghanistan, from FATA and other associated regions due to the Floods and regional conflicts. Low response rate in this district may be due to prevalence of resistance types common in other regions.

In FATA regions, the lowest response was found which again could be attributed to the prevalence of resistant HCV genotypes that may have resulted due to the influx of people from the Gulf and Central Asian Countries.

## Conclusion

The above discussion shows that antiviral therapy against HCV infection in chronic HCV patients of KPK province is 74.71%. The high response rate may be due to the prevalence of genotypes 2 and 3.

## Competing interests

The authors declare that they have no competing interests.

## Authors' contributions

SA (research scholar) carried out sampling and experimental work, BA supervised the research work conducted by SA and designed the experimental work and manuscript preparation with the help of IA (Co-Supervisor). SA helped in manuscript reviewing and corrections prepared by research scholar. The final manuscript is approved by all of the authors after reviewing it critically.
